# Supplementary material for: Is the C-Terminal Domain an Effective and Selective Target for the Design of Hsp90 Inhibitors against Candida Yeast?
Source: Microorganisms. 2023 Nov 22;11(12):2837. doi: 10.3390/microorganisms11122837 (PMC10745388; doi:10.3390/microorganisms11122837)
Supplement: Supplementary file 1 [file microorganisms-11-02837-s001.zip › Supplementary Table S1.pdf]

**Supplementary Table S1.** Hsp90 inhibitors in clinical trial since 2005 and referenced in ClinicalTrials.gov on the May 10th, 2021.

| Design | Class* | Drug name | Phase | Company | Number of trials completed or ongoing |
|---|---|---|---|---|---|
| Natural product based inhibitors | | | | | |
| | Radicicol derivatives (resorcinol analogues) | | | | 75 |
| | | STA-9090 (Ganetespib) | I, II & III | Synta Pharmaceuticals and Aldeyra Therapeutics | 37 |
| | | NVP-AUY922 (Luminespib) | I & II | Cancer Research Technology, The Institute of Cancer Research (UK) and Vernalis | 24 |
| | | AT13387 (Onalespib) | I & II | Astex Pharmaceuticals | 12 |
| | | KW-2478 | I & II | Kyowa Hakko | 2 |
| | Geldanamycin derivatives | | | | 57 |
| | | 17-AAG (Tanespimycin KOS-953 CNF-1010) | I, II & III | National Institutes of Health (USA) and Kosan Biosciences | 37 |
| | | IPI-504 (Retaspimycin) | I, II & III | Infinity Pharmaceuticals | 12 |
| | | 17-DMAG (Alvespimycin) | I & II | National Cancer Institute (USA) and Kosan Biosciences | 6 |
| | | IPI-493 | I | Infinity Pharmaceuticals | 2 |
| Rational drug designed synthetic inhibitors | | | | | |
| | Purine-scaffold derivatives | | | | 17 |
| | | CNF2024 (BIIB021) | I & II | Biogen | 7 |
| | | PU-H71 (Zelavespib) | I | Memorial Sloan-Kettering Cancer Center and Samus Therapeutics | 5 |
| | | Debio 0932 (CUDC-305) | I | Curis | 3 |
| | | BIIB028 | I | Memorial Sloan-Kettering Cancer Center and Biogen | 1 |
| | | MPC-3100 | I | Myriad Pharmaceuticals | 1 |
| | Benzamide derivatives | | | | 13 |
| | | SNX-5422 | I & II | Serenex and Esanex | 12 |
| | | TAS-116 (Pimitespib) | I | Taiho Pharmaceutical | 1 |
| | Tropane-containing derivatives | | | | 4 |
| | | XL888 | I | Exelixis | 4 |
| | Pyrimidine derivatives | | | | 2 |
| | | HSP990 | I | Vernalis | 2 |
| | Monoclonal antibody | | | | 1 |
| | | Mycograb (Efungumab) | I | NeuTec Pharma Ltd | 1 |
| | Undisclosed | | | | 2 |
| | | DS-2248 | I | Daiichi Sankyo Inc | 1 |
| | | TQB3474 | I | Chia Tai Tianqing Pharmaceutical Group | 1 |

*Except Mycograb which bind the MD of yeast and human Hsp90, all the inhibitors in trials target the ATP-NTD of human Hsp90.
