# Supplementary material for: Is the C-Terminal Domain an Effective and Selective Target for the Design of Hsp90 Inhibitors against Candida Yeast?
Source: Microorganisms. 2023 Nov 22;11(12):2837. doi: 10.3390/microorganisms11122837 (PMC10745388; doi:10.3390/microorganisms11122837)
Supplement: Supplementary file 1 [file microorganisms-11-02837-s001.zip › Supplementary Table S2.pdf]

Supplementary Table S2. Hsp90 inhibitors already tested against the yeast *Candida* and their activity alone *in vitro* and in combination *in vitro* or in *Galleria mellonella* model according to literature data.

Compounds	Candida species	Activity alone	Activity in combination	References
**NTD-Hsp90 inhibitors**				
**RA**	*C. albicans*<br>*C. glabrata*	MIC 8 - >20 μM	-Synergistic activity with fluconazole<br>-Diminution of tolerance to voriconazole<br>-Diminution of the micafungin paradoxical effect<br>-Diminution of caspofungin resistance	[25-27]
**STA9090**	*C. albicans*	IC_{50} > 40 μM	-Weak synergistic activity with fluconazole	[27,28]
**NVP-AUY922**	*C. albicans*	IC_{50} > 40 μM	-Weak synergistic activity with fluconazole	[27,28]
**GA**	The five most common species*	IC_{50} 5.7-22.8 μM	-Synergistic activity with triazoles and echinocandins	[29-31]
**17-AAG**	*C. albicans*	No indicated	-Synergistic effect with fluconazole in *G. mellonella*	[32]
**17-DMAG**	*C. albicans*	No indicated	-Synergistic effect with fluconazole in *G. mellonella*	[32]
**SNX-2112**	*C. albicans*	IC_{50} > 40 μM	No indicated	[27]
**HSP990**	The five most common species*	IC_{80} > 168 μM	-Synergistic activity with fluconazole	[28]
**CTD-Hsp90 inhibitors**				
**SIL**	*C. albicans*<br>*C. krusei*<br>*C. tropicalis*	MIC 2 mM	-Antagonistic effect with nystatin	[33]
**EGCG**	The five most common species*	MIC 8.5-34.1 μM	-Synergistic effect with azoles	[34,35]

*The five most common species are *C. albicans, C. glabrata, C. parapsilosis, C. tropicalis* and *C. krusei*
